# Isolation and genomic characterization of bacteriophage Vanta, a lytic *Yuavirus* infecting clinical strains of *Pseudomonas aeruginosa*

**DOI:** 10.1128/mra.00466-25

**Published:** 2026-07-31

**Authors:** Aaryan Harshith, Zachary N. Flamholz, Paul L. Bollyky, Jessica C. Sacher

**Affiliations:** 1 Division of Infectious Diseases, Stanford Department of Medicine, Stanford University 6429, Stanford, California, USA; DOE Joint Genome Institute, Berkeley, California, USA

**Keywords:** *Pseudomonas*, phage, AMR, novel, antimicrobial, cystic fibrosis

## Abstract

We report the genome of Vanta, a lytic bacteriophage isolated from wastewater using *Pseudomonas aeruginosa* PA14. Phage Vanta is a *Yuavirus* with a circularly permuted, 61,663-bp, 64.5% guanine-cytosine, dsDNA genome. Head width is 56 ± 2 nm, and tail length is 133 ± 5 nm. Vanta plaques on two drug-resistant clinical *P. aeruginosa* isolates.

## ANNOUNCEMENT

*Pseudomonas aeruginosa* is an opportunistic gram-negative pathogen causing skin, lung, and bone infections worldwide (1, 2). Antimicrobial resistance has made it a target for bacteriophage therapy (3, 4). Here, we report the genome of Vanta, a lytic, tailed *P. aeruginosa* phage.

**Fig 1:**
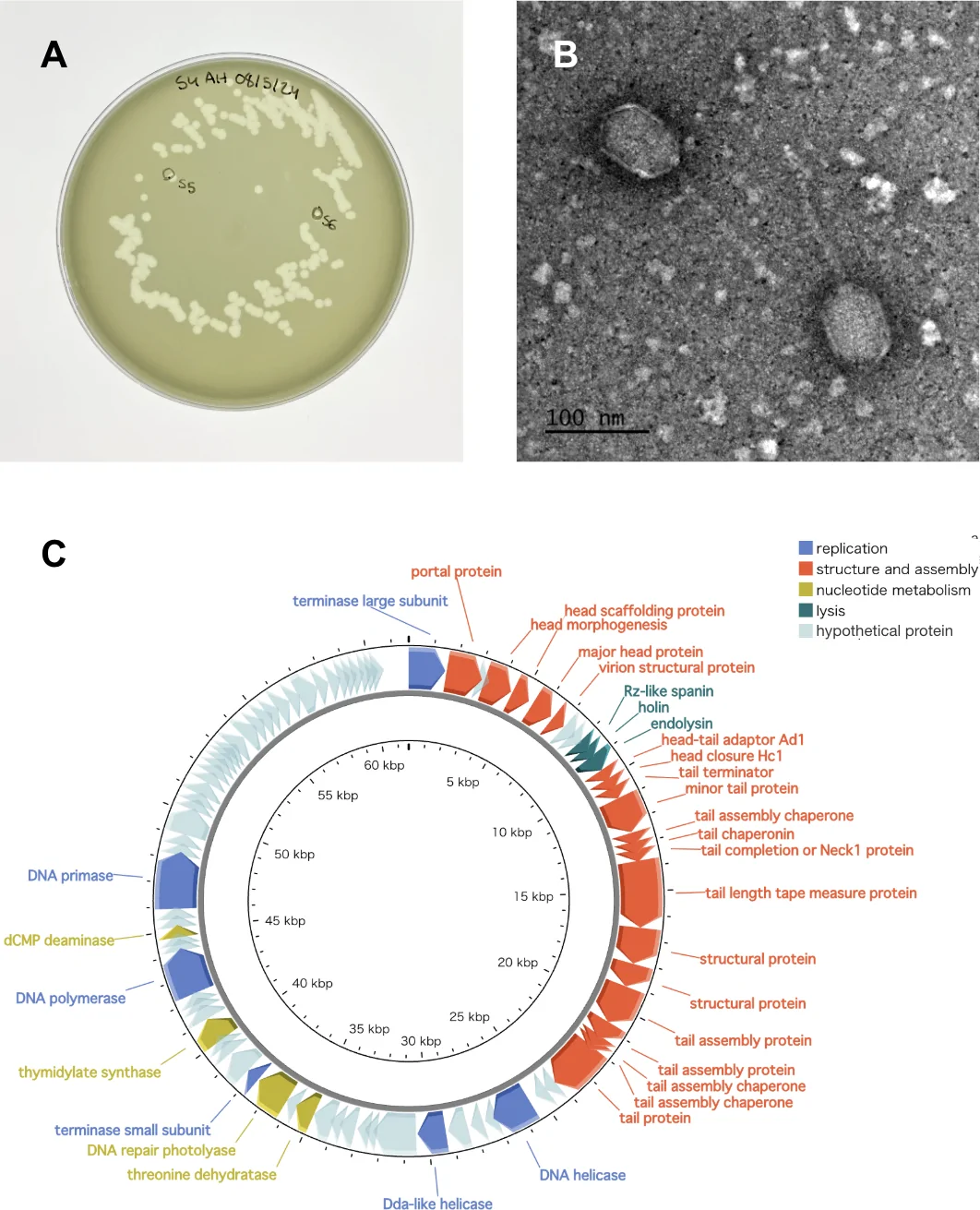
(**A**) Plaques formed by Vanta on *P. aeruginosa* PA14. (**B**) TEM of Vanta. (**C**) Predicted circularized genome map generated using Proksee 2023 (5).

Vanta was isolated from wastewater collected on 11 July 2024 from Stanford University’s Codiga Resource Recovery Center. Wastewater was diluted in saline magnesium (SM) buffer (100 mM NaCl, 8 mM MgSO₄, 50 mM Tris-HCl, 0.01% gelatin), sterile-filtered (0.2-µm polyethersulfone), and enriched for phages using early-log *Pa* PA14 grown in LB Miller broth (200 µL wastewater, 5 mL culture, 37°C, 250 rpm). After 4 h, the culture was centrifuged twice (15 min, 5,000× *g*, 4°C). Sterile-filtered supernatant was applied to a PA14 lawn. A round, transparent, 2-mm plaque was transferred into 1 mL SM buffer using a P1000 pipette tip. To ensure an isolated phage, plaque picking was repeated 5× sequentially before propagating (Fig. 1A).

Vanta plaqued on *Pa* mPAO1Δpf4Δpf6 (6), *Pa* Li010, and two multidrug-resistant *Pa* strains from rectal and bronchial infections (CDC AR Isolate Bank AR_0231, AR_0238) (7).

Vanta was negatively stained (1% uranyl acetate) and visualized at 120 kV using a JEM1400 transmission electron microscope (JEOL, Tokyo, Japan), which showed a flexible, non-contractile tail typical of siphoviruses. Mean head width and tail length were 56 ± 2 and 133 ± 5 nm, respectively (*n* = 10, mean ± standard deviation [SD], Fig. 1B).

Filtered lysate was treated with DNAse (2 U/mL), RNAse A (10 µg/mL), and proteinase K (30 µg/mL) (NEB, Ipswich, USA), and DNA was extracted using a DNeasy kit (Qiagen, Germantown, USA). Whole-genome sequencing (WGS) libraries were prepared (Novogene Standard Microbial WGS PCR+ Kit) and sequenced (Illumina NovaSeq X Plus Series PE150, Novogene, Sacramento, USA). Next, 1.1 × 10^7^ paired-end reads (150 bp) were verified with FastQC (v0.12.1) (8). Adaptors and low-quality reads (≤100 bp, FastQ ≤20) were removed with BBDuk (v39.08) (9). Reads were subsampled to 80× with Seqtk v1.4 (10) and assembled using MEGAHIT (v1.2.9) (11). Contigs were visualized using Bandage (v0.8.1) (12) and validated using BBMap (v38.84) with 100% read coverage (31,851 mapped reads) (13). A single 61,663-bp contig (64.5% guanine-cytosine [GC]) was extracted (Fig. 1C) and polished using Pilon (v1.24) (14). PhageTerm (v1.0.12) suggested the genome was circularly permuted and complete (15, 16).

The genome was oriented to the terminase large subunit gene using Geneious Prime (v.2026.0.2) (5, 17) and annotated using Pharokka (v1.8.2) (18), with Prodigal (v2.6.3) for gene prediction (19), PHROGs for functional annotation (20), and tRNAscan (v2.0.12) for tRNA identification (21). Next, 86 coding sequences and 0 tRNAs were identified. BACPHLIP (v0.9.6) predicted Vanta was lytic (98% confidence) (22).

Phylogeny.fr (v2.0) classified Vanta into the genus *Yuavirus* using FastTree (v2.2.0) (23, 24), which was confirmed with VIPTree (v4.0) using phage AAT1 as an outgroup (25).

All tools were run with default parameters unless specified.

## Data Availability

The annotated genome of Vanta is accessible through NCBI GenBank (PQ628237). Raw reads are also available (BioProject PRJNA1267729, SRA SRR36199512).
